# Chitosan Combined with ZnO, TiO_2_ and Ag Nanoparticles for Antimicrobial Wound Healing Applications: A Mini Review of the Research Trends

**DOI:** 10.3390/polym9010021

**Published:** 2017-01-09

**Authors:** Vu Khac Hoang Bui, Duckshin Park, Young-Chul Lee

**Affiliations:** 1 Department of BioNano Technology, Gachon University, 1342 Seongnamdaero, Sujeong-gu, Seongnam-si 13120, Gyeonggi-do, Korea; hoangvu210190@gmail.com; 2 Korea Railroad Research Institute (KRRI), 176 Cheoldobakmulkwan-ro, Uiwang-si 16105, Gyeonggi-do, Korea; dspark@krri.re.kr

**Keywords:** chitosan, metal (oxide) nanoparticles, nanocomposites, antimicrobial ability, wound healing

## Abstract

Chitosan is a natural polymer that has been widely utilized for many purposes in the food, textile, agriculture, water treatment, cosmetic and pharmaceutical industries. Based on its characteristics, including biodegradability, non-toxicity and antimicrobial properties, it has been employed effectively in wound healing applications. Importantly, however, it is necessary to improve chitosan’s capacities by combination with zinc oxide (ZnO), titanium dioxide (TiO_2_) and silver (Ag) nanoparticles (NPs). In this review of many of the latest research papers, we take a closer look at the antibacterial effectiveness of chitosan combined with ZnO, TiO_2_ and Ag NPs and also evaluate the specific wound healing application potentials.

## 1. Introduction

Despite the helpful developments in medical and pharmaceutical technology, harmful bacteria, infecting millions of people annually, remain a great concern. The United States spends more than 120 billion USD per year for the treatment of infectious diseases, five billion USD of which is earmarked exclusively for the treatment of resistant pathogens [1]. Research into new antibiotics is not of interest to many large pharmaceutical companies, due to the facts that it is time consuming, expensive (around one billion USD annually) and risky, not to mention the short commercial life of such drugs (due to resistance acquisition by bacteria). Nonetheless, the rise of resistant pathogens coupled with the significant decrease in the rate of antibacterial-agent approval in recent decades has made the battle with bacterial infections one of the greatest health challenges facing the world [2]. More attention and resources must be devoted to finding smart solutions to this problem that are both inexpensive and effective. Recent “bottom-up” approaches based in nanotechnology could help.

Nanocomposites (NCs) are the second generation in nanotechnology, which refers to the assemblies of hetero- or homo-nanoparticles structures for different purposes; this combination not only enhances the properties of independent NPs in the mixture, but also reveals new functionalities [3]. There is also a trend to combine metal or metal oxide with natural polymers to enhance the antimicrobial ability. For example, the antibacterial activity of chitosan incorporated with Ag NPs is higher than that of each component [4]; or the presence of AgNO_3_, which is added to PVA/CS blend solutions, can improve not only the antibacterial activity, but also the electrospinning ability [5].

Chitosan is derived from chitin: the second most common natural polysaccharide. Chitin can be obtained from many sources, such as insect exoskeletons, arthropod shells, such as crustaceans (e.g., shrimp, prawn, crabs), and cephalopod beaks, as well as fungi cell walls [6]. Chitosan exhibits many promising biological activities, consisting of antimicrobial activity, antitumor activity, hemostatic activity and wound healing acceleration [7,8]. Chitosan, with its unique biological characteristics, including biodegradability, non-toxicity and antimicrobial functionalities, has been widely applied in industries ranging from foods to textiles, agriculture, water treatment, cosmetics and pharmaceuticals [9]. Chitosan rather than any other natural compound is the main focus of this review owing to its free amino groups and correspondingly unique capacity to combine strongly with metal ions [10,11]. There are two explanatory models for the structural connection of chitosan to metal ions: the “pendant model”, where only one amino acid group of chitosan is bound to one ion, and the “bridge model”, where several nitrogen atoms, hydroxyl groups or even more than one chitosan chain are all bound to one ion [12,13].

## 2. Antimicrobial Properties of Chitosan

**Figure 1:**
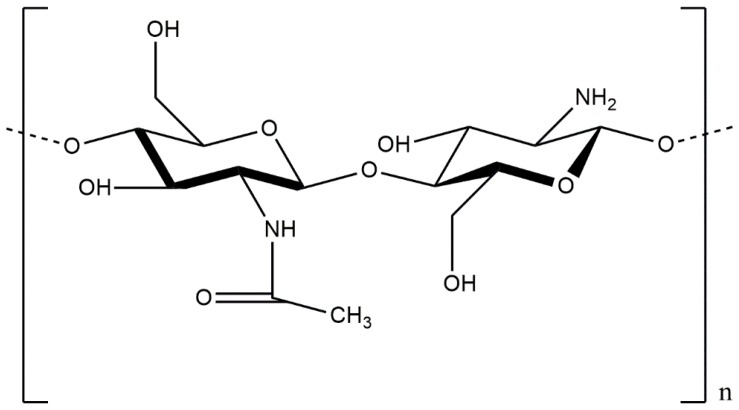
Structure of chitosan.

**Figure 2:**
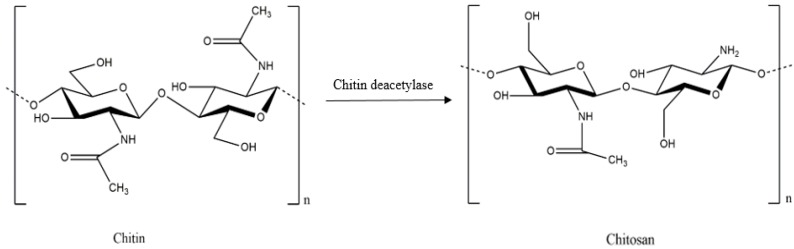
Deacetylation of chitin to chitosan.

Chitosan is a linear polysaccharide composed of randomly-distributed β-(1-4)-linked d-glucosamine and *N*-acetyl-d-glucosamine, similarly to cellulose (Figure 1). A common method for the synthesis of chitosan entails removal of an acetyl moiety from chitin through hydration or enzymatic hydrolysis in the presence of chitin deacetylase (Figure 2) [14,15].

Chitosan’s and its derivatives’ mechanisms of antimicrobial activity are believed to be similar to other cationic biocidals, following six steps: (1) bacterial cell surface adsorption; (2) cell wall diffusion; (3) cytoplasmic membrane adsorption; (4) cytoplasmic membrane disruption; (5) cytoplasmic constituents leakage; and (6) cell death [16].

Chitosan’s inhibitory efficiency against different microorganisms is the subject of considerable debate. In some reports, its antimicrobial activity is stronger against Gram-negative bacteria than Gram-positive [17,18], while in another study, it is better against Gram-positive bacteria, due to the structure of the outer membrane barrier of Gram-negative bacteria [19]. Still other studies have found no significant differences in chitosan’s antimicrobial activity between Gram-positive and Gram-negative bacteria [20]. These contradictory results reflect differences among the studies’ initial reaction materials and experimental conditions [9]. Chitosan also has antifungal and anti-yeast activities, and due to a combination of factors, including increasing of solubility, chitosan may be more antimicrobial at reduced pH values. For example, in study of Roller and Covill (1999), chitosan glutamate has more inhibitory against *Mucor racemosus* at a pH of 4.5 than pH 5.2 [21].

Important determinants of chitosan’s antibacterial activity are bacterial cell-surface characteristics. In their structures, bacterial species are complex and heterogeneous. Surface appendages, such as pili, fimbriae or flagella, or surface polymers, such as lipopolysaccharide (LPS), mycolic acids, lipoteichoic acid (LTA), capsular polysaccharides or proteins, vary significantly [22]. These appendages and polymers can strongly attach to antibacterial agent surfaces in short-range interactions, such as hydrogen bonding [23]. In fact, even polymers can span over relatively long distances and affect attachment, even in cases where cells do not experience any net attraction [24].

Chitosan also can adhere with bacteria-cell-surface polyanions through electrostatic interaction. Gram-negative bacteria are more absorbed to chitosan and have a higher inhibitory effect compared with Gram-positive bacteria due to the higher negative charge on the cell surface [17]. Strand et al. (2002), in testing the efficiency of chitosan of various compositions applied for flocculation of different bacteria, found that the purely electrostatic interactions may not act as the primary role in Gram-negative bacteria flocculation and that, instead, cell-surface hydrophobic forces affecting non-electrostatic interactions are a more important factor in bacteria and surface interaction.

The antibacterial mechanism of chitosan begins with the interaction with the cell surface and the compromising of the outer membrane. At a pH below p*K*a, polycations of chitosan compete with divalent metals for binding with polyanions that compose the cell surface. However, at a pH above p*K*a, the activity switches to chelation. The cell wall is then likely to lose its integrity or the activity of degradative enzymes will be affected, due to Mg^2+^ and Ca^2+^ ions’ replacement in the cell wall [9].

Due to cell wall damage, the cell membrane is unprotected. Its permeability therefore will be drastically altered due to the contact between chitosan and its bilayer, and the surface charge of bacteria will be promptly neutralized and even reversed [25,26]. Increased membrane permeability leads to cell membrane destabilization, intracellular substances’ leakage and, in serious cases, cell death [9]. There are six steps in the wound healing process, consisting of inflammation, migration of the cell, angiogenesis, synthesis of provisional matrix, deposition of collagen and re-epithelization [27]. Chitin and chitosan have the abilities to enhance the wound healing process. The repeating mono-subunit, which is present in chitin and chitosan, *N*-acetyl glucosamine (NAG), is an important component of dermal tissue and necessary for scar tissue repair [28]. Chitin and chitosan effectively support cell growth by their high positive surface charge [29] and their surface leads to thrombosis and blood coagulation [30]. The chitosan membrane surface has free amino groups, which may complex with acidic groups of the blood cells [27].

Due to the great concern about the over-use of antibiotics leading to drug-resistant bacterial strains, there is a growing trend to replace them with alternative materials in wound healing applications. Among the many candidates, chitosan has been considered greatly, due to its biodegradability, non-toxicity and antimicrobial properties. However, the loose cationic nature and poor solubility of chitosan at a pH above 6.5 limits it in practical applications. To overcome this problem, one strategy is the modification of the backbone chain of chitosan. Modified chitosan also enhanced the antimicrobial activity [31]. Chitosan derivatives that are highlighted in the literature include: quaternized chitosan [32], carboxyalkylated chitosan [33], sulfonated and sulfobenzoyl chitosan [34,35], carbohydrate-branched chitosan [36] and chitosan-amino acid conjugation [37].

Another strategy to enhance the properties of chitosan is its combination with other metal (oxide) NPs [38]. Fortunately, among the many natural compounds, chitosan, due to its free amino groups, strongly complexes with other metals or metal oxide NPs. Additionally, among the many metals (oxides), ZnO, TiO_2_ and, especially, Ag NPs have been considered for combination with chitosan in many studies.

In order to fabricate wound-dressing products, chitosan commonly needs to be incorporated with another polymer. Careful selection of a suitable polymer and processing method will determine the success of the final product. Most of the research noted in this review has considered materials of low- or non-toxicity to humans and animals; nonetheless, to maintain durability, stability and, thus, prevent their release into the environment, a method to effectively immobilize nanocomposites on various polymer surfaces is required.

## 3. Nanotechnology in Antimicrobial Wound-Dressing Applications

According to Archana et al. (2013), an advantageous wound dressing should have the following characteristics: (1) an appropriate water vapor transmission rate (WVTR) to produce a humid environment on the wound bed, prevent the risk of dehydration and exudate accumulation; (2) adequate gas permeability for the processing of oxygen-requiring repair; (3) removal of excessive bacterial-nutrient-containing exudates from the wound bed by providing a high fluid adsorption capability; (4) an effective barrier against the infection of harmful bacteria; (5) antibacterial activity beneath the dressing for suppression of bacteria growth; and (6) the lack of any cytotoxic effects if there is secondary damage to the newborn tissue [27]. The risk of antimicrobial infection is one of the most important factors that must be considered when evaluating wound healing materials.

Between different methods, broth dilution and radial diffusion are frequently used for determining the antimicrobial activity of wound healing nanocomposite materials. One common broth dilution method is ASTM-2149, which was described by Petkova et al. (2014). Briefly, harmful bacteria were pre-cultured in appropriate broth culture to reach ~10^8^ CFU/mL, then pieces of nanocomposite materials were added to 5 mL of the bacteria suspension. Before introducing the wound healing material with bacteria and after 15, 30 and 60 min, the suspension was withdrawn and diluted in sterile buffer solution, placed on agar and counting the bacterial colony forming unit after 24 h of cultivation at 37 °C [12]. For radial diffusion methods, as regards Archana et al. (2013), an agar Petri dish was spread with harmful bacteria at a concentration of ~10^8^ CFU/mL, and the wound healing materials were placed. These dishes then were cultivated at 37 °C for 12 h, then the inhibition zone was measured consequently [27]. Between the two types of methods, the broth dilution method directly measures the survival of bacteria after contact with wound healing materials, while in the agar diffusion method, the antimicrobial ability of the materials was defined indirectly by the diameter of the surrounding inhibition zone.

There are already many chitin- and chitosan-based wound dressings available on the market [39]. Chitosan, notwithstanding its high healing abilities and use as a single antimicrobial agent in many wound-dressing studies, needs to be combined with nanomaterials for improved hemostatic performance, healing capacity and application flexibility.

### 3.1. Nanocomposite Materials Based on Chitosan and ZnO NPs

#### 3.1.1. Antimicrobial Properties of ZnO NPs

Another wound healing application trend is the use of nanocomposite combinations of chitosan and zinc oxide (ZnO) NPs. ZnO is a promising material with wide applicability based on its characteristics, including distinct optical, chemical sensing, semiconducting, electric conductivity, as well as piezo-electric properties [40]. Synthesis of ZnO NPs, obtainable by many different methods, affords products of varying size and morphology [41]. Methods that have been used for the synthesis of ZnO NPs in the literature are microwave decomposition, the simple wet chemical route, the deposition process, the simple precipitation method, hydrothermal synthesis, the solvothermal method, the microwave hydrothermal method and the hydrothermal technique. ZnO NPs, as reported in the literature, exhibit strong activities against different kinds of bacteria. The photocatalytic generation of hydrogen peroxide has been reported for the main antimicrobial mechanism of ZnO NPs [42]. Furthermore, it has been reasonably well established that bacterial growth inhibition by contact with ZnO is affected by penetration and consequent disorganization of the cell membrane [43,44]. The role of particle size on the antibacterial behavior of ZnO NPs has been the subject of debate: Jones et al. (2008) showed that smaller ZnO NPs have higher toxicity [45]; Franklin et al. (2007), however, found that the antimicrobial abilities of ZnO NPs are not affected by their size [46]. Besides high antimicrobial activity, ZnO NPs possess high optical absorption capacities in the regions of UVA (315–400 nm) and UVB (280–315 nm); these abilities make ZnO NPs advantageous in the manufacturing of cosmetics that combine UV protection with antimicrobial functionality [47].

Antimicrobial tests usually are conducted in watery or cell culture media. Unfortunately, due to the high polarity of water leading to deposition, ZnO NPs agglomerate with water almost during synthesis. The synthesis process in fact is impeded by agglomeration, re-precipitation, settling or non-dissolution. A number of studies have sought to tackle this problem by the addition of different kinds of polymer, such as poly(vinyl alcohol) (PVA), poly(vinyl pyrrolidone) (PVP), poly(α,γ,l-glutamic acid) (PGA) or poly(ethylene glycol) (PEG), which can enhance ZnO morphology and size without any significant side effect on antibacterial effectiveness [41,48]. Another research focus has been the utilization of appropriate capping agents or deflocculates, such as sodium silicate (Na_2_SiO_3_) or sodium carbonate (Na_2_CO_3_), for the same above-noted purpose [49].

A number of studies has evaluated ZnO NPs’ minimum inhibition concentration (MIC) and minimum bactericidal concentration (MBC). Emami-Karvani and Chehrazi (2011), using agar diffusion methods, recorded MIC values for *Escherichia coli* and *Staphylococcus aureus* of 3.1 and 1.5 mg/mL, respectively [50]. These figures, notably, are nearly the same as those reported by Reedy et al. (2007): 3.4 mg/mL for *E. coli* and 1 mg/mL for *S. aureus* [51]. Both research teams agreed that Gram-positive bacteria are more vulnerable than Gram-negative bacteria to ZnO NPs, based on their differences in physiology, constitution and metabolism of the cell and cell wall [52,53]. Xie et al. (2011) found that, as measured against other bacteria, the MIC of 30-nm ZnO NPs to *Campylobacter jejuni* was around 0.05–0.025 mg/mL, 8–16-fold lower than *E. coli* O157:H7 and *S. enterica* serovar *Enteritidis* (0.4 mg/mL) [54]. In the recent report of Salem et al. (2015), ZnO NPs’ most effective concentration against *E. coli* and *Vibrio cholerae* was between 1.6 × 10^5^ and 1.2 × 10^6^ particles/mL, while that for silver Ag NPs was 1.2 × 10^7^ particles/mL [55].

The main mechanism of ZnO NPs’ antibacterial activity is the functionality of reactive oxygen species (ROS) [56,57,58,59]. ZnO NPs, after sufficient photo absorption, promote electron transfer from the valence band to the conduction band, leaving a charged hole in the valence band. The electrons are free to travel within the conduction band. An adjacent molecule will fill the hole by a migrated electron, leaving it with a hole. These electrons’ and holes’ reaction yields ROS, such as •O_2_^−^ and •OH, whose reactive agents therefore can react to produce H_2_O_2_, •OH and •OOH. The mechanism of ROS generation can be expressed by the following chemical equations [41]:

ZnO + hv → e^−^ + h^+^

h^+^ + H_2_O → •OH + H^+^

e^−^ + O_2_ → •O_2_^−^

•O_2_ + H^+^ → •OH_2_

•OH_2_ + H^+^ + e^−^ → H_2_O_2_

Raghupathi et al. (2011) showed that increased ROS production by ZnO NPs under UV exposure enhances the antibacterial utility of ZnO NPs [60]. The toxicity of ROS, such as superoxide anion radical (•O_2_^−^), hydrogen peroxide (H_2_O_2_) and hydroxide radical (•OH^−^), leads, via penetration into the bacterial cell membrane, to the destruction of lipids, deoxyribonucleic acid (DNA) and proteins, as well as other components. Padmavathy and Vijayaghavan (2008) confirmed the release of ROS from the ZnO NPs under both UV and visible light [49]. Due to their negative charge, the superoxide and hydroxyl radicals cannot enter the bacterial membrane and are found on the outer surface [54]. The H_2_O_2_ molecules, on the contrary, have the ability to enter the cell wall of bacteria, leading to damage and destruction of the cell cytoplasm and, thereby, accelerated cell death [61,62]. Further, when ZnO NPs are present in the growth media, they will continue releasing peroxides, eventually covering entire dead bacteria surfaces. This continuous release of peroxide affords higher bactericidal efficacy [41].

Another suggested antimicrobial mechanism of ZnO NPs is the zinc ions’ (Zn^2+^) release in the media [63,64,65,66,67]. The released Zn^2+^ damage bacteria by active transport inhibition, amino acid metabolism and enzyme system disruption [41]. Two main parameters affecting the release of Zn^2+^ were identified by Pasquet et al. (2011): (1) the particles’ physicochemical properties, consisting of porosity, concentration, size and morphology; (2) the elements of the media, such as pH, UV illumination, exposure time and the presence of others. The role of zinc ion release continues to be debated [68]. Kasemets et al. (2009), studying the toxicity of ZnO NPs to *Saccharomyces cerevisiae*, implicated the solubility of Zn^2+^ in bacterial-containing solution. They also suggested that only Zn^2+^ can induce a relatively high tolerance in bacteria at solubilized low concentrations [69]. On the other hand, based on the results of Sawai (2003) and Jiang et al. (2009), due to the low concentration Zn^2+^ released from dissolution of ZnO, they suggested that the distribution of Zn^2+^ with respect to the inhibition of microorganisms of ZnO NPs is limited [42,70]. ZnO NPs themselves are insoluble in water; unless they are capped or stabilized, the Zn^2+^ release into the medium is impeded, and thus, also, their antimicrobial activity is curtailed [67].

Some research has suggested that the direct interaction between NPs and the bacterial membrane can lead to cell death. Specifically, it has been posited that the precipitation of NPs on the exterior of bacteria, or their accumulation in the cytoplasmic or periplasm space, can affect membrane disturbance and disorder [43,61]. Zhang et al. (2008) and Stoimenov et al. (2002) reported that electrostatic forces are induced by bacterial treatment with ZnO NPs and that this electrostatic interaction between NPs and bacterial cell surfaces can retard bacterial growth [59,71]. It is inevitable that, due to the excessive formation of separated carboxyl groups, total bacteria will carry a negative charge, while ZnO NPs are positively charged in water suspension; the result is membrane damage followed by the NPs’ internalization into the cells [59]. Brayner et al. (2006) observed the internalization of NPs into cells after cell wall disorganization resulting from interaction between *E. coli* and ZnO NPs. Their scanning electron microscope (SEM) images showed the change in ZnO NPs, both inside and outside the cell membrane, probably as the result of the bacteria’s lipopolysaccharide release [43]. The same phenomena-ZnO NPs attaching to the cell wall, entering inside and leading to disruption and consequent disorder and leakage of cell components, were observed also by both Xie et al. (2011) and Diaz-Visurraga et al. (2011) [54,72].

Regarding the toxicity effects of ZnO nanoparticles to human and animal cells, Sharma et al. (2008) tested ZnO NPs’ cytotoxicity against human epidermal cells. There is an increase of Olive tail moment of 2.13 ± 0.12 (0.8 µg/mL) of ZnO NPs treated samples compared with 1.37 ± 0.12 of control sample after 6 h in the Comet assay. ZnO NPs also showed glutathione (59% and 51%), catalase (64% and 55%) and superoxide dismutase (72% and 75%) depletion at concentrations of 0.8 and 0.08 µg/m. ZnO NPs were also found to have DNA damage potential and oxidative stress induction in cells. Their data indicated that, even at a low concentration, ZnO NPs hold cytotoxicity potential toward human epidermal cells. They suggested that care should be taken when using ZnO NPs in their handling and especially in dermatological preparations [73].

However, Hanley et al. (2008), when testing the normal human cells’ response to ZnO NPs and comparing it to cancer cells; response under various signaling environments, found that ZnO NPs displayed a higher potential to destroy cancerous T cells than normal cells. Compared to normal cells, the mechanism of toxicity of T cells produced higher inducible levels. Besides, ZnO NPs accelerated apoptosis induction and reactive oxygen species inhibition, which led to cell death. Their finding indicated a potential application of ZnO NPs in cancer treatment or autoimmunity [74].

#### 3.1.2. Applications of Chitosan/ZnO Nanocomposites in Wound Healing

Kumar et al. (2012) introduced a bandage composed of composite chitosan hydrogel and ZnO NPs (CZBs). Their antimicrobial test indicated that this bandage had strong effects on *E. coli* and *S. aureus*, with a higher antimicrobial impact on *E. coli*. Further, in vivo wound healing evaluations showed that the CZBs, as compared with Kaltosat, a chitosan control and a bare wound, had a high wound-dressing utility with no toxicity [38]. Vicentini et al. (2009), following the Pechini method, incorporated ZnO NPs into blend films of chitosan, poly(vinyl alcohol) (PVA) and Tween 80 (T80), the results revealing that the ZnO NPs and T80 influenced the film properties. Specifically, X-ray diffraction (XRD) and FITR investigations revealed the reduction of the intra- and inter-molecular hydrogen bond lengths and greater tensile strength and elongation at break, respectively. The blend films also indicated a porous morphology, due to the degeneration of H_2_O_2_ forming oxygen and water, which was proportional to the increase of the T80 concentration. Increased degeneration and swelling, moreover, was demonstrated through experiments with Hank’s solution. Finally, an antimicrobial test against *S. aureus* confirmed the role of ZnO NPs in the blend films [75]. Samzadeh-Kermani and Miri (2014) also used ZnO NPs and chitosan, specifically by grafting them with polyaniline and montmorillonite. Their bactericidal experiments against *S. aureus* and *E. coli*, by the paper-disc diffusion method, showed that the 1.0% and 1.5% ZnO NPs concentrations had strong activities compared with composite samples of higher or lower concentrations, due to decreased cell membrane interaction, leading to inhibition zone reduction [76].

Petkova et al. (2014) introduced cotton fabrics generated by simultaneous sonochemical deposition of ZnO NPs and chitosan. The processing method, entailing 30-min sonochemical coating and 2 mM ZnO NPs suspension, resulted in the relatively high antibacterial inhibition against *E. coli* and *S. aureus*. When depositing the same concentration of chitosan with ZnO NPs, the samples illustrated 48% and 17% higher antibacterial activity against the target species. Even after several washings, the samples retained their durability, showing 21% and 40% improvements for *E. coli* and *S. aureus*, respectively. Finally, the hybrid ZnO/chitosan coating, tested against fabrics coated with ZnO NPs alone, showed an 87% fibroblast-biocompatibility improvement compared with a steady decrease of cell viability for the latter over the course of one week [12].

Karahaliloglu et al. (2016) recently introduced wound dressing with the chitosan/silk sericin scaffolds combined with lauric acid (LA) and ZnO NPs. The presence of the individual components was confirmed by Fourier transform infrared photoacoustic spectra (FTIR) and energy-dispersive X-ray spectroscopy-(EDX). By SEM observations, scaffolds have an interconnected microporous structure, and there is no effect on pore size and porosity by the reinforcement of ZnO NPs or LA. While compare chitosan/silk sericin/ZnO NPs with chitosan/silk sericin/LA, against *Escherichia coli*, the inhibition zone extended from 2 ± 0.4–7 ± 0.1 mm, while against *Staphylococcus aureus*, these figure increased from 2.5 ± 0.2–6 ± 0.4 mm. Besides the high antimicrobial activity against Gram-positive and Gram-negative bacteria, scaffolds also revealed increased human keratinocyte (HaCaT) cell proliferation and viability [77].

### 3.2. Nanocomposites Based on Chitosan and TiO_2_ NPs

#### 3.2.1. Antimicrobial Properties of TiO_2_ NPs

According to Foster et al.’s (2011) thorough review, titanium dioxide (TiO_2_) is an effective semiconductor and photocatalytic material. There are many ways to synthesize TiO_2_ nanomaterials, including sol-gel, micelle and inverse micelle, sol, hydrothermal, solvothermal, direct oxidation, chemical vapor deposition, physical vapor deposition, electrodeposition, sonochemical method and microwave methods [78]. There are three main types of TiO_2_ polymorphs: anatase, rutile and brookite. Among them, most researchers have used the anatase and rutile phase. However, due to the difference in the extent of the recombination of electrons and holes between the two, anatase has been the more effective photocatalyst [79]. Some studies, meanwhile, have shown the combination of anatase with rutile or brookite to be a more effective photocatalyst than anatase alone [79,80,81]. The interactions between the two forms of TiO_2_ lead to the reduction of bulk recombination and, thus, it is assumed, to increased photocatalytic activity [82]. Because UVA irradiation is required to activate TiO_2_, the indoor use of TiO_2_ is limited and needs to be modified to work with visible light. Fujishima and Zhang (2006) combined TiO_2_ with C, N, S and metals, like Sn, Pd and Cu, as well as dyes. The modified catalyst could reduce the band gap, so photocatalysis can be activated in the range of visible light; the problem, though, was that their anti-microbial activity was lower than that with UVA alone [83]. This strategy, the just-noted problem notwithstanding, remains the main subject of much research.

Some studies have shown that changes in cell permeability caused the death of bacteria cells while interacting with TiO_2_. The rapid leakage of K^+^, followed by the slow release of RNA and protein, has been observed in many studies [84,85,86]. Huang et al. (2000) suggested an increase in the permeability of the membrane of *E. coli* after being exposed with TiO_2_ due the increased permeability of small molecules, such as *ο*-nitrophenol β-d-galactopyranoside, and the leakage of large molecules, such as β-d-galactosidase [87].

Microscopic changes have been noted in many studies as confirmation of cell wall and membrane change after the treatment of bacteria cells with TiO_2_. Amezaga-Madrid et al. (2002, 2003) demonstrated, by SEM and Transmission electron microscopy (TEM) observations, *Pseudomonas aeruginosa* membrane-structural changes, such as “bubble-like protuberances expelling cellular material”, and suggested that the bubbles might have been due to localized damage to the peptidoglycan layer and resultant extrusion of the inner membrane [88,89].

Bacterial cell membrane damage also has been confirmed in several studies by the production of membrane breakdown products. In the report of Maness et al. (1999), the release of malondialdehyde demonstrated lipid peroxidation by ROS, and the reduction of 2,3,5-triphenyltetrazolium chloride defined the loss of membrane respiratory activity [90].

DNA is an exception among the many components damaged by TiO_2_ penetration of bacterial cells. Comet assay data from Varghese and Foster (2011) showed a lack of any DNA damage even when 97% of bacteria was killed. They also suggested that whereas damage to DNA by TiO_2_ also has been shown in many studies, this might come late, after membrane disruption and cell death [82].

In most studies, ROS is mainly responsible for bacteria death. The mechanism of ROS production is similar to ZnO NPs. The scavengers play important roles in killing bacteria. Salih et al. (2002) abolished, by the presence of dimethyl sulfoxide (DMSO) and cysteamine, the enhancing effects of TiO_2_, thus suggesting the involvement of •OH in the cell-killing process [91]. However, •OH exists for a short period and tends to not diffuse more than 1 µm from the TiO_2_ surface, especially in the presence of organic matter. Kikuchi et al. (1997) found that *E. coli* was killed even when a porous membrane with a 50-µm thickness was used to separate it from the TiO_2_ surface. They also observed that H_2_O_2_ killed *E. coli* both with and without a membrane. On the basis of these results, they suggested that both •OH and H_2_O_2_ take part in the killing of bacteria, with H_2_O_2_ acting at a distance [92]. However, according to Guillard et al. (2008), there was no antibacterial activity when a dialysis membrane was used to separate *E. coli* from TiO_2_ [93]. It has been considered that, in the presence of ferrous ions, H_2_O_2_ acts at a distance by producing •OH via the Fenton reaction [82]:

Fe^3+^ + •O^2−^ → Fe^2+^ + O_2_

Fe^2+^ + H_2_O_2_ → Fe^3+^ + OH^−^ + •OH

For the toxicity of TiO_2_ against human and animal cells, Ghosh et al. (2012) evaluated the toxic effect of commercial TiO_2_ NPs through cytotoxic, genotoxic, hemolytic and morphological observation. Against human lymphocyte cells, the cytotoxic effects of TiO_2_ NPs were attributed to the damage of the membrane, mitochondria, metabolic activity and the stability of the lysosomal membrane. Lymphocyte cells’ genotoxicity was measured by the Comet assay, then PI/Annexin V staining was used to evaluate the mechanism of cell death (apoptosis/necrosis). TiO_2_ NPs were also examined for their hemolytic characteristic, osmotic fragility and hemoglobin interaction. Atomic force microscopy (AFM) was used for morphological studies of the alteration of human erythrocyte cells. Their data suggested that TiO_2_ NPs could significantly decrease the activity of mitochondrial dehydrogenase and induce DNA damage and apoptosis in human lymphocyte cells. However, the integrity of the membrane was not affected by the treatment. Through characterization by spherocytosis and echinocytosis, human erythrocyte cells also displayed a hemolytic property of TiO_2_ NPs. The interaction between TiO_2_ NPs and hemoglobin was discovered by spectral analysis. By their toxic potential, these authors suggested that the use of commercial TiO_2_ NPs should be cautioned [94].

Saquib et al. (2012) tested the effect of TiO_2_ NPs on the cytotoxicity and DNA damage of human amnion epithelial (WISH: Wistar Institute, Susan Hayflick) cells. In the concentration range of 0.625–10 µg/mL and through the MTT assay, TiO_2_ NPs showed potential cytotoxicity effects. TiO_2_ NPs also showed a significant decrease in catalase activity and glutathione level. As compared to the control samples, intracellular ROS generation showed a 1.87-fold increase, and G2/M cell cycle arrest showed a 7.3% increase. At a concentration of 20 µg/mL TiO_2_ NPs, the DNA double-strand formation was shown to be broken with a 14.6-fold higher Olive tail moment (OTM) value in contrast to control samples. Consequently, TiO_2_ NPs indicated potential cyto- and geno-toxicity against WISH cells [95].

Recently, Kongseng et al. (2016) examined the TiO_2_ NPs’ cytotoxicity against peripheral blood mononuclear cells (PBMCs). After 24 h of treatment at a concentration ≥25 µg/mL, TiO_2_ NPs decreased cell viability and toxic mediator products, as well as inflammatory response cytokines were increased. There was also an induction in cell apoptosis. At a TiO_2_ NP concentration ≥125 µg/mL, cyclooxygenase-2 and interleukin-1β were significantly expressed. Their data indicated that TiO_2_ NPs have cytotoxicity towards human blood cells [96].

#### 3.2.2. Applications of Chitosan/TiO_2_ Nanocomposites in Wound Healing

The combination of chitosan with TiO_2_ is a strategy to enhance the wound healing effectiveness of chitosan. Dressing materials based on TiO_2_ play the role of support platforms for the adhesion and growth of bone and stem cells and, meanwhile, the control of hemorrhage by enhanced blood clotting [97,98]. Jayakumar et al. (2011) used the lyophilization technique to introduce chitin-chitosan/TiO_2_ NP composite scaffolds. Their results showed that the composite TiO_2_ NPs decreased the scaffold pore size. XRD and TGA studies, meanwhile, displayed that the composite scaffolds were amorphous and had a higher thermal stability than conventional ones. As for the FITR studies, they showed no chemical changes. However, these authors suggested that the addition of TiO_2_ NPs, while decreasing the pore size as noted above, also reduced the swelling degradation. Moreover, there was no cytotoxicity toward an array of cell lines, including osteoblast-like cells (MG-63), fibroblast cells (L929) and human mesenchymal stem cells (hMSCs) [99].

Archana et al. (2013) introduced a ternary nano-dressing consisting of TiO_2_-NP-loaded chitosan-pectin. The chitosan-pectin formation is the result of the electrostatic attractions between the ionized carboxyl acid groups (COO^−^) of pectin and the ionized amino groups of chitosan (NH_3_^+^). In that study, SEM measurement showed a broad, 20–40-nm particle size distribution of TiO_2_ NPs in the matrix. The morphological study also revealed that the TiO_2_ NPs were well distributed in the resulting material. With the incorporation of TiO_2_ NPs and the decrease of the pectin content (1:1), the tensile strength of the dressing was increased from 12.6 ± 1.0–14.28 ± 1.0 MPa. The agar diffusion method indicated high antibacterial activity against different bacteria. With the concentration of bacterial culture around 10^8^ CFU/mL, the inhibition zones for *Escherichia coli*, *Staphylococcus aureus*, *Pseudomonas aeruginosa*, *Bacillus subtilis* and *Aspergillus niger* were 45, 45, 47, 49 and 29 mm, respectively. The nanomaterial could induce blood coagulation and showed good hemostatic properties, as well as no cytotoxicity toward L929 or NIH3T3 mouse fibroblast cells. Further, in an in vivo wound healing study on injured rats, the chitosan-pectin-TiO_2_, compared with a chitosan-treated group and gauze dressing only, healed faster, demonstrating 99.01% closure after just 16 days [27].

Woo et al. (2015) fabricated wound dressing with an upper layer of TiO_2_ NPs combined with a chitosan membrane and a sub-layer of human adipose-derived extracellular matrix (ECM) sheet as the sub-layer. The purpose of the dense and fibrous top layer is the protection of the wound from bacterial infection, while the goal of the sponge-like sub-layer is to accelerate new tissue regeneration. In antimicrobial test, there was a 33.9% and a 69.6% decrease in *E. coli* and *S. aureus* viability by using a modified drop plate method. The bilayer composites have good biocompatibility and provided proper physiochemical and compositional cues at the wound site in in vivo experiments using rats. Compared to the control sample and through changes in histological examination and wound size, the resulting wound dressing yields faster regeneration of granulation tissue and epidermis with less scar formation [100].

### 3.3. Nanocomposites Based on Chitosan and Ag NPs

#### 3.3.1. Antimicrobial Properties of Ag NPs

Since ancient times, the bactericidal effects of Ag have been observed. The recent improvement of nanotechnology by “bottom-up” approaches has led to the design of several types of Ag NPs with different and tunable physico-chemical properties (e.g., size, shape and surface chemistry) [101]. Ag NPs have been widely utilized for different purposes, including diagnosis, treatment, drug delivery, medical-device coating, wound dressing, medical textiles and contraceptive devices. Successful synthesis and use of Ag NPs in different fields and antimicrobial applications has been reported by a huge and ever-expanding literature [102]. Rizzello and Pompa (2014) suggested that the absence of NP standard assays and of any definitive explanation of their molecular mechanisms of action are the key issues [102].

Ag NPs are synthesized via physical, chemical and biological methods. In physical synthesis, laser ablation and evaporation/condensation are the common methods. In the evaporation or condensation technique, a furnace tube is used to produce Ag NPs under atmospheric pressure, while in the laser synthesis technique, a laser is used to ablate metals in solution without chemical agents, and Ag nano-colloids could be obtained [103,104,105].

**Figure 3:**
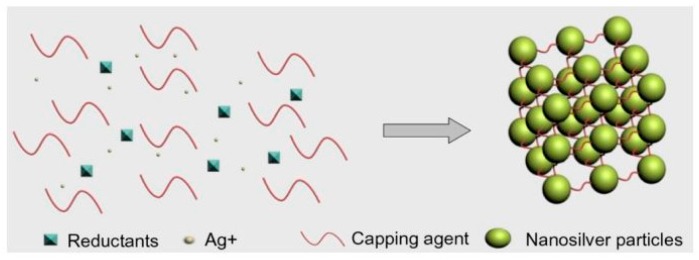
Chemical synthesis of Ag NPs. Reproduced with permission from Dovepress, 2014 [105].

In chemical synthesis, the most frequently-applied method is chemical reduction. In this method, silver salt, reductants and a stabilizer or capping agents are used as the three main elements to manage the growth of Ag NPs. Due to its being more inexpensive and having greater stability compared with other silver salts, AgNO_3_ is often used for Ag NP production, while the common reductants are borohydride, citrate, ascorbate and hydrogen gas [106,107,108,109]. The common stabilizers are surfactants and ligands or polymers, such as polyvinyl pyrrolidone, poly(ethylene glycol), poly(methacrylic acid), as well as poly(methyl methacrylate), and others (Figure 3) [105].

**Figure 4:**
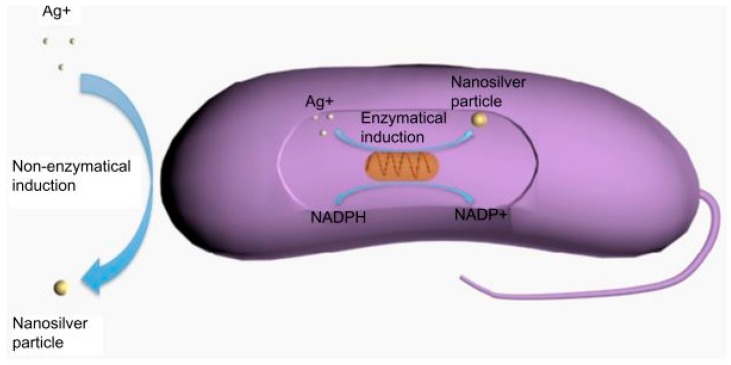
Biological synthesis of Ag NPs. Reproduced with permission from Dovepress, 2014 [105].

In biological synthesis, protein, carbohydrate, bacteria, fungi, yeast, algae and plants can be used as the reducing agents and stabilizers where organic solvents and toxic regents are absent [110,111,112,113,114]. The two possible mechanisms of biological synthesis are enzymatic and non-enzymatic reduction [115]. The enzymatic reduction of Ag NPs could be via nicotinamide adenine dinucleotide phosphate-dependent reductase; while in non-enzymatic reduction, this is similar to chemical reduction, with microorganisms or plants being used as the reducing and stabilizing agents (Figure 4) [112].

The antibacterial utility of Ag NPs depends on different parameters, including particle shape, size and concentration. Among the variable shapes of Ag material are Ag nanoplates, nanorods and NPs. Sadeghi et al. (2012) and Pal et al. (2007) found that Ag nanoplates had the best antibacterial activity [116,117]. Additionally, although Pal et al. (2007) were not able to elucidate the exact role of Ag nanoplates in high antibacterial activity, they assumed that it is related to their positive surface charge, which enhances electrostatic interactions with bacterial cells [117].

Size is one of the factors that affects the antimicrobial ability of Ag NPs. The results of studies by Sotiriou and Pratsinis (2010) and Morones et al. (2005) found that smaller NPs (<10 nm) more easily attach to the surfaces of cell membranes than do larger NPs and, thereby, have shown more antibacterial activity [118,119].

In a comparison of Ag NPs with other Ag materials, such as AgCl colloids and Ag^+^ ions, Choi and Hu (2008) discovered that the Ag NPs were the most efficient (the EC_50_ were 0.14, 0.25 and 0.27 mg/L, respectively). They also confirmed that the smallest Ag NPs have stronger efficacy than the larger ones [120].

While Ag NPs have wide-ranging effects on a broad spectrum of Gram-negative, Gram-positive and even antibiotic-resistant bacteria, the operative antibacterial mechanism remains only partially understood. However, it is widely accepted that Ag NPs can adhere to and enter into the bacterial cell wall, causing cell membrane structural and permeability alterations, thus leading, in serious cases, to cell death. Another probable mechanism entails that the formation of ROS species can damage cell membranes. Ag NPs also can release Ag^+^ that contacts with many important enzymes and phosphorus-containing bases through thiol groups, thereby inhibiting certain critical functions, such as the division of cells or the replication of DNA. Moreover, through changes to the phosphotyrosine profiles of bacterial peptides, signal transduction can be modulated [121,122,123,124].

Some studies have demonstrated Ag NP cytotoxicity to animal cells. In the review of Ge et al. (2014) [105], whereas the exact toxicity mechanism is still unclear, they suggested that Ag NPs are ionized in the cells, resulting in the activation of ion channels; cell membrane permeability is changed to both potassium and sodium, and mitochondrial interaction and the apoptosis pathway are induced via ROS production, which leads to cell death [125,126,127]. In a recent investigation, Kim et al. (2008) measured the oral toxicity of various doses of 60 nm Ag NPs to Sprague-Dawley rats for 28 days. After the testing period, there were minute, but different, changes in bodyweight between male and female rats. Furthermore, neither the micronucleated polychromatic erythrocytes, nor the ratio of polychromatic erythrocytes to total erythrocytes differed between the rats exposed to Ag NPs and the control rats. They suggested that the Ag NPs in fact did not induce genetic toxicity in rats. However, slight liver damage was shown in the rats exposed to more than 300 mg of Ag NPs by the change of alkaline phosphate and cholesterol, and a dose-dependent accumulation of Ag NPs was observed in all of the tissues [128]. Lee et al. (2007) imaged the transport and biocompatibility of single Ag NPs over a wide diameter (5–46 nm) range in an early-development zebrafish embryo study. They found that Ag NPs could enter in and out of the embryos through chorion pore canals (CPCs), but became trapped inside the CPCs and the inner mass of the embryos. They observed Ag NPs that have biocompatibility and cytotoxicity by transport inside embryos at different development stages and in normally developed, deformed, as well as dead zebrafish. These effects were highly dependent on the Ag NPs concentration, the critical dosage being 0.19 nM [129]. Contrastingly, according to the good review by Lansdown (2007), even though Ag_2_S deposits have been found in the cutaneous nerve region, there was no evidence proving that the peripheral nervous system was toxically affected by the Ag NPs. Although Ag_2_S was seen in the blood-brain and blood-cerebrospinal fluid (CSF) barrier tissues or was deposited in basement membranes or collagen, there was no cytotoxicity effect [129]. Ji et al. (2007), having conducted an in vivo inhalation toxicity test on eight-week-old Sprague-Dawley rats for 28 days, found no different changes in body weight, hematology or biochemical values relative to the Ag NP dose [130].

DiVicenzo et al. (1985), having demonstrated the difficulty of complete Ag NP removal from animal and human tissues once already deposited in the body, nonetheless observed Ag NP release through the hair, urine and feces [131]. With most cytotoxicity investigations conducted in in vitro experiments with human and animal cells, along with short time experiments in animal, the toxicity of Ag NPs to human and animal cells continues to be debated. Although the toxicity to human and animal cells is not clear over the long term, it is recommended that care be exercised when applying Ag NPs to different areas of the body.

#### 3.3.2. Applications of Chitosan/Ag Nanocomposites in Wound Healing

The combination of Ag NPs with chitosan for the enhancement of antimicrobial utility has been much discussed in the literature. Chitosan has the ability to stabilize the shape of Ag NPs [132]. The methods employed for the fabrication of Ag NPs, in most cases, have been electrospinning-based, due to the rapid, efficient, simple and inexpensive nanofiber production thus enabled [133]. Chitosan cannot be produced straightforwardly by an electrospinning method, owing to its polycationic nature in solution. To overcome this obstacle, many researchers have mixed chitosan with other polymers. Among the various types of polymers utilized, poly(vinyl alcohol) (PVA) has proven popular due to its good biocompatibility, biodegradability, mechanical characteristics and, especially, its fiber- and film-forming utility, which facilitates nanofiber fabrication [5]. Hang et al. (2010) introduced non-woven mats of a poly(vinyl alcohol) (PVA)/chitosan (CS) and PVA/CS combined with Ag NPs by an electrospinning method. Their physiochemical characterization experiments showed the average diameter of electrospinning fibers in a PVA non-woven mat containing Ag NPs to be around 577.1 nm. The addition of AgNO_3_ to PVA/CS solution, moreover, demonstrably increased the electrospinning activity of the PVA/CS blend solution; indeed, the morphology observation of electrospinning Ag/PVA/CS fibers was “no bead” and “uniform fiber structure” at a high chitosan concentration in the solutions. The Ag NPs were found to play the role of a nucleating agent during cold crystallization; their sizes were 2.44 nm, 6.05 nm, 6.78 nm and 10.74 nm according to the contents of chitosan in the PVA/CS blends: 0%, 4%, 5.5% and 12%, respectively. Overall, the chitosan in the PVA/CS blend solutions effected a reduction in the diameters of the electrospinning fibers and an improvement in the tensile strength, but with a decrease in the elongation. An antimicrobial test of *E. coli* revealed a higher activity for non-woven Ag/PVA/CS than for PVA/CS. The Ag/PVA/CS non-woven mats only showed anti-activity at a bacteria concentration around 7 × 10^6^ CFU/mL [5]. In Abdelgawad et al.’s (2012) research group, Ag NPs were reduced with glucose and mixed with chitosan. PVA solution was combined with the chitosan/Ag NPs at different ratios, and the final nanofiber was derived through electrospinning and glutaraldehyde cross-linking. No beading was noticed in the mats of the PVA/CS-Ag NPs at weight ratios of 95/5, 90/10, 85/15 and 80/20, whereas beading occurred at 70/30, 60/40 and 50/50. However, fibers of 60/40 PVA/CS-Ag NPs still maintained good uniformity even when beaded structures were observed. The antibacterial experiments on *E. coli* showed that samples with initial concentrations of 7 × 10^5^ CFU/mL and 7 × 10^7^ CFU/mL were totally killed when interacting with PVA/CS 80/20 and 60/40 fiber mats. Still though, some bacterial colonies were observed after the application of fibers with a reduced chitosan loading (blend ratio: 90/10) for both bacterial concentrations. The antibacterial utility of the composite PVA/CS-Ag NP fiber mats were enhanced by the loading of Ag NPs. The PVA/CS-Ag NP fiber mats with 20% or a higher concentration of chitosan had bactericidal effects, while those with lower chitosan content only had bacteriostatic effects against *E. coli*. These results confirmed that the presence of Ag NPs, besides enhancing electrospinning performance, also improves the antibacterial effectiveness of nanofiber [134].

Ong et al. (2008) produced a chitosan composite wound dressing with polyphosphate and Ag NPs. The result indicated that optimal formation of the chitosan-polyphosphate dressing, relative to the chitosan-only alternative, could accelerate blood clotting, increase platelet adhesion, generate thrombin faster and absorbed more blood. Ag-loaded chitosan-polyphosphate dressing was significantly higher in bacterial activity than the chitosan-polyphosphate one in vitro and completely killed *P. aeruginosa* and *S. aureus*. A significant reduction of *P. aeruginosa*—from 90%–14.3%—also was shown in a mice wound-infection model [135]. Lu et al. (2008), having fabricated composite Ag NPs/chitosan (CS) films, utilized sterility and pyrogen tests to ensure wound dressing biosafety. In the results, the Ag NPs/CS dressing showed an increased rate of wound healing, and the association with the Ag NPs levels in blood and tissues appeared to be lower than with the silver sulfadiazine (C_10_H_9_AgN_4_O_2_S) dressing (SSD). Specifically, on Day 13, the healing rate of the Ag NPs/chitosan dressing group was 98.98% ± 6.09% compared with 81.67% ± 6.30% for the SSD group. The healing time of the Ag NPs/CS dressing group was 3.94 days shorter than that of the SSD group. The blood-Ag concentrations of the Ag NPs/CS dressing group were always lower than those of the SSD group, redundant and returned to normal after 13 days of treatment. On the 45th day of treatment, the Ag contents in liver, kidney and brain increased in both the Ag NPs/CS and SSD groups, though in the latter group, the liver-Ag content was 100-times higher than normal [136].

Sponges composed of chitosan-hyaluronic acid (HA)/Ag NPs were introduced by Anisha et al. (2013). HA was used, because it is a glycosaminoglycan, a major component of the skin extracellular matrix, and has good hydrophilic, as well as unique viscoelastic properties. The nanocomposite sponges were prepared by homogeneous mixing of chitosan, HA and Ag NPs followed by freeze drying to obtain a flexible and porous structure. HA takes part in wound healing inflammation, granulation tissue formation, re-epithelialization and re-modeling. It also has been shown to have a positive effect on scarless wound healing. Although sponges with higher Ag NP concentrations (0.005%, 0.01% and 0.02%, respectively) have a significant effect on reducing the growth of *E. coli*, *S. aureus*, *P. aeruginosa*, *Klebsiella pneumonia* and methicillin-resistant *S. aureus*, cytotoxicity and cell attachment studies have shown that Ag NPs concentration-dependent toxicity to fibroblasts might present an obstacle to the practical clinical use of composite sponges [111].

Celebi et al. (2013) developed a nanofiber with chitosan (CS)/poly(vinyl alcohol) (PVA)-containing Ag^+^ incorporated with hydroxyapatite (HAP) particles. The aim of using HAP was to control the release of ions in order to improve the durability of nanofibers. Uniform fibers of 60–70 nm in diameter were achieved throughout the electrospinning process with a CS/PVA ratio of 25/75, a flow rate of 0.01 mL/min and a spinning distance of 10 cm. CS/PVA nanofibers containing 0.5 and 1.0 wt % Ag^+^-HAP were shown to have clear effects on *E. coli* in antimicrobial tests [137]. Hebeish et al. (2014) prepared a nanocomposite with chitosan-grafted-poly acrylonitrile Ag nanocomposites (CS-g-PAN/Ag). Ag NPs of an average 15–20-nm diameter were dispersed homogeneously in a CS-g-PAN/Ag nanocomposite-ray according to UV spectra and TEM images. The inhibition zones, 16 nm with *E. coli* and 15 nm with *S. aureus* in antimicrobial tests, indicated the effectiveness of the nanocomposite [138]. Thomas et al. (2012) synthesized film with chitosan and Ag NPs by photochemical methods, specifically by reducing Ag^+^ in an acidic solution of AgNO_3_ and chitosan. The resultant thin film showed excellent antibacterial activities against *E. coli* and *B. subtilis*. For example, according to the viable cell count method, the CFU number of *E. coli* on a nutrient plate treated with a suspension containing plain chitosan discs (80 CFU/cm^2^) was much larger than that on a plate with a suspension of Ag NP-loaded chitosan discs (20 CFU/cm^2^). These trends were replicated with experiments against *B. subtilis*, in which the figures for the plate with a suspension containing plain chitosan discs and that with a suspension of Ag NP-loaded chitosan film were 50 CFU/cm^2^ and 8 CFU/cm^2^, respectively [139].

Li et al. (2010) prepared films with chitosan, Ag and ZnO NPs through sol-cast transformation. By this method, Ag NPs were generated using chitosan as the reducing agent under the hot alkaline condition, and ZnO NPs were formed in the composite at the same time. An antimicrobial test based on the agar plate method was used against seven strains, including *S. aureus*, *E. coli*, *B. subtilis*, *Penicillium*, *Aspergillus*, *Rhizopus* and yeast. Although the CS-8 sample (with 0.5 wt % Ag and 10% ZnO) demonstrated excellent efficiency, the CS-4 sample (with 0.1 wt % Ag and 10 wt % ZnO) exhibited a better overall potential for real-world application, due to the former’s imperfections in terms of blended film color and higher Ag toxicity [140].

In comparison to other shapes, a stronger antimicrobial effect was observed in Ag NPs, which have truncated triangular or hexagonal shapes displaying the {111} face plane [117,141]. Chitosan films that are incorporated with hexagonal Ag NPs were developed and characterized by Levi-Polyachenko et al. (2016). Hexagonal Ag NPs are non-cytotoxic at low concentrations and have an elevated temperature generation ability. The number of viable peripheral blood mononuclear, keratinocytes or fibroblasts cells on the film was either maintained or increased while applying the composite film. Differential scanning calorimetry (DSC) and Fourier transform infrared photoacoustic spectra (FTIR-PAS) experiments showed no significantly differences in the film structure synthesized with hexagonally- or spherically-shaped Ag NPs. Hexagonal Ag NPs were also found to be advantages for mild hyperthermia generation and fluorescently-labeled dextran intracellular delivery. The appearance of hexagonal Ag NPs in chitosan wound dressing showed that they could induce the proliferation of cells, protect against the infection of bacteria and generated mild hyperthermia generation for small molecules delivery [142].

## 4. Conclusions

Due to its biodegradability, non-toxicity and antimicrobial properties, chitosan has been considered to replace traditional materials in wound healing applications. However, chitosan loses its cationic nature and solubility in an alkaline environment. Besides modifying its backbone chain, the properties of chitosan could be enhanced through strong complexation with other metals (oxide) by free amino groups. Among many metals and metal oxides, ZnO, TiO_2_ and Ag NPs are attractive candidates in combination with chitosan. Regarding the antimicrobial behavior between three nanomaterials, Ag NPs seems to have higher antimicrobial ability, but not always [55,143,144,145]. Because each material is variable in size and shape, these comparisons still need to be investigated further in the future. The combination of ZnO, TiO_2_ and Ag NPs with chitosan not only improved antimicrobial activity, but also accelerated the wound healing process and enhanced the mechanical characteristics of wound materials (Table 1). However, the cytotoxicity of these composite materials to human and animal cells, especially in long time frames, is still unclear and delays their full implementation.

Due to their ready suitability for combination with different kinds of materials (besides the metal and metal oxides noted in this review), chitosan and its derivatives can also be combined with bacterial cellulose, alginate or graphene [146,147,148]. Our laboratory is now working on a method for the fabrication of chitosan with water-soluble and cationic aminopropyl magnesium phyllosilicate (AMP), which exhibits little or no cytotoxic effect and that has already been proven to have a high antimicrobial capacity. The antimicrobial ability of AMP clays is distributed to their amino propyl groups. When the AMP clay was absorbed into the bacterial inner membrane, its amino propyl group contacted the negatively-charged lipid membrane due to electrostatic interaction. These interactions therefore lead to the disruption of the tighter lipid bilayer, accelerated membrane function events, the increase of membrane permeability and end with bacterial content leakage [149]. If this endeavor is successful, we will have another means of utilizing chitosan nanocomposites in wound healing and medical applications.

## Figures and Tables

**Table 1:** Summary of chitosan combined with ZnO, TiO_2_ and Ag NPs for antimicrobial wound healing applications.

Authors	Materials	Products	Remarkable Results
Kumar et al. (2012) [38]	Chitosan + ZnO NPs	Bandages	High antimicrobial activity against *E. coli* and *S. aureus*. Good swelling, blood clotting ability. No cytotoxicity on normal human dermal fibroblast (nHDF) cells and fast wound healing process.
Vicentini et al. (2009) [75]	Chitosan + ZnO NPs + poly(vinyl alcohol) + Tween 80	Films	Compared to chitosan/PVA: chitosan/PVA/ZnO NPs have higher thermal stability; the reduction of tensile strength and elongation at break reduced; the degradation and swelling ratio increased; and they have stronger antimicrobial activity against *S. aureus*.
Samzadeh-Kermani and Miri (2014) [76]	Chitosan + polyaniline + montmorillonite + ZnO NPs	Films	High antimicrobial activity against *S. aureus* and *E. coli*.
Petkova et al. (2014) [12]	Chitosan + ZnO NPs	Textiles	High antimicrobial activity against *S. aureus* and *E. coli*. Chitosan + ZnO NPs showed 87% improvement in biocompatibility, and cell viability was steady decreased after 1 week.
Karahaliloglu et al. (2016) [77]	Chitosan + ZnO NPs + silk sericin	Scaffolds	Higher antimicrobial activity against *E. coli* and *S. aureus* and increased HaCaT cells’ proliferation and viability when compared with chitosan/silk sericin/acid lauric.
Jayakumar et al. (2011) [99]	Chitin/chitosan + TiO_2_ NPs	Scaffolds	The presence of TiO_2_ NPs increases thermal stability and decreases pore size and swelling degradation. No cytotoxicity on an array of MG-63, fibroblast cells (L929) and human mesenchymal stem cells (hMSCs).
Archana et al. (2013) [27]	Chitosan + pectin + TiO_2_ NPs	Films	High antimicrobial activity against a wide spectrum of bacteria. The presence of TiO_2_ NPs increased tensile strength, induced blood coagulation, good hemostatic ability, no toxicity on L929 and NIH3T3 fibroblast cells and faster healing.
Woo et al. (2015) [100]	Chitosan + TiO_2_ NPs	Bilayer composite	High antimicrobial activity, proper physiochemical, good biocompatibility and faster wound healing.
Hang et al. (2010) [5]	Chitosan + poly(vinyl alcohol) + Ag NPs	Fiber mats	The presence of Ag NPs increased electrospinning activity, showed no beads and a uniform fiber structure. Higher antimicrobial against *E. coli* when compared with non-Ag NP mats.
Abdelgawad et al. (2012) [134]	Chitosan + poly(vinyl alcohol) + Ag NPs	Fiber mats	The presence of Ag NPs improved electrospinnability, decreased the diameter of fibers and enhanced antimicrobial activity against *E. coli*.
Ong et al. (2008) [135]	Chitosan + polyphosphate + Ag NPs	Films	The presence of Ag NPs increased antimicrobial activity against *Pseudomonas aeruginosa* and *Staphylococcus aureus* when compared to chitosan/polyphosphate films.
Lu et al. (2008) [136]	Chitosan + Ag NPs	Films	The presence of Ag NPs increased the wound healing process; silver content in rat organs was lower than silver sulfadiazine.
Anisha et al. (2013) [111]	Chitosan + poly(vinyl alcohol) + Ag NPs	Sponges	The presence of Ag NPs lowered the growth of a wide spectrum of harmful bacteria. Higher concentration of Ag NPs leads to the reduction of fibroblast cell viability.
Celebi et al. (2013) [137]	Chitosan + poly(vinyl alcohol) + hydroxyapatite	Fiber mats	No growth of *E. coli* was observed.
Hebeish et al. (2014) [138]	Chitosan + poly acrylonitrile + Ag NPs	Graft nanocomposite	High antimicrobial activity against *E. coli*.
Thomas et al. (2012) [139]	Chitosan + Ag NPs	Films	The presence of Ag NPs increased the antimicrobial ability against *E. coli* and *B. subtilis*.
Li et al. (2010) [140]	Chitosan + Ag NPs + ZnO NPs	Films	Chitosan/Ag NPs/ZnO NPs have high antimicrobial activity against a wide range of spectrum bacteria and stronger than chitosan/Ag NPs and chitosan/ZnO NP films.
Levi-Polyachenko et al. (2016) [142]	Chitosan + Ag NPs	Films	The number of peripheral blood mononuclear, keratinocyte and fibroblast cells was maintained or increased when contacted with chitosan/Ag NP films. The presence of Ag NPs induced cell proliferation, increased antimicrobial activity and generated mild hyperthermia for the delivery of small molecules.
